# Investigation of preoperative physical activity level in kidney transplant recipients and its impact on early postoperative recovery: A retrospective cohort study

**DOI:** 10.3389/fsurg.2022.1062652

**Published:** 2023-01-06

**Authors:** Guo Li, Qi-fan Guo, Shang-ping Zhao, Miao-wei Wang, Xia Zhang, Ao Wang, Chen-fan Gui, Qi-ling Tan, Qiang Gao

**Affiliations:** ^1^ Department of Rehabilitation Medicine, West China Hospital, Sichuan University, Chengdu, People's Rublic of China; ^2^ Key Laboratory of Rehabilitation Medicine in Sichuan Province, West China Hospital, Sichuan University, Chengdu, China; ^3^ Centre of Kidney Transplantation, West China Hospital, Sichuan University/West China School of Nursing, Sichuan University, Chengdu, China

**Keywords:** kidney transplantation, recipient, physical activity, early mobilization, early postoperative recovery

## Abstract

**Objective:**

To retrospectively investigate the preoperative physical activity (PA) level in kidney transplant recipients (KTRs) and its impact on early postoperative recovery.

**Methods:**

A total of 113 patients who received kidney transplantation at West China Hospital of Sichuan University were enrolled in this retrospective cohort study. According to the PA level measured by the Chinese version of the International Physical Activity Questionnaire—Long Version, the patients were allocated into the low PA level group (Group L, *n* = 55) and medium to high PA level group (Group MH, *n* = 58). The kidney function recovery indicators, including estimated glomerular filtration rate (eGFR), postoperative complications, postoperative length of stay (LOS), and unscheduled readmission within three months of discharge, were evaluated and documented. A association analysis was applied to analyze and compare the association between indicators.

**Results:**

The median PA levels of the KTRs were 1701.0 MTEs * min/week. Regarding the postoperative recovery indicators, the KTRs spent a mean time of 19.63 h to achieve transfer out of bed after the operation (Group L: 19.67 h; Group MH: 19.53 h; *P* = 0.952) and reached a mean distance of 183.10 m as the best ambulatory training score within two days after the operation (Group L: 134.91 m; Group MH: 228.79 m; *P* < 0.001). The preoperative PA level showed a moderate positive association with early postoperative ambulation distance (*ρ* = 0.497, *P* < 0.001). However, no significant between-group difference in eGFR on postoperative days 1, 3, and 5 (*P* = 0.913, 0.335, and 0.524) or postoperative complications, including DGF (*P* = 0.436), infection (*P* = 0.479), postoperative LOS (*P* = 0.103), and unscheduled readmission (*P* = 0.698), was found.

**Conclusions:**

The preoperative PA level of KTRs is lower than that of the general population. KTRs with moderate or high preoperative PA levels showed higher ambulatory function in the early postoperative period than those with low preoperative PA levels, but no between-group differences in other early recovery indicators were observed.

## Introduction

Kidney transplantation is the optimal choice for patients with end-stage renal disease (ESRD) to improve their physiological function and quality of life (QoL). However, due to the limited source of donated kidneys and other barriers, only a few patients are lucky enough to receive kidney transplantation. More than 10,000 kidney transplantations occur in China annually, while over 300,000 patients are still on the waiting list with a very high ratio of 30:1 (1). When waiting for transplantation, patients may face increasing risks of mortality and morbidity caused by frailty and loss of physiological function (2). Even in the posttransplant period, kidney transplant recipients (KTRs) still face increased risks of dysfunction, prolonged hospital stays, unscheduled readmission, low levels of QoL, and graft morbidity caused by the abovementioned reasons (3, 4). A low physical activity (PA) level is an important symbol of frailty and dysfunction, while physical activity is also globally recommended for people across different countries, environments, communities, and populations (5, 6). Therefore, in patients with chronic kidney disease (CKD) during dialysis or posttransplantation, it is suggested that they participate in feasible exercise programs to achieve a higher PA level and reduce health risks (7).

The PA level is considered a modifiable risk factor among KTRs to reduce the incidence of cardiovascular disease (CVD), which is the leading cause of graft loss, morbidity, and mortality. Prolonged low PA levels have been suggest to be a risk factor for complications among ESRD patients (8), such as CVD, diabetes, and sarcopenia. In addition, previous studies have reported that KTRs generally suffer from low PA levels both during the preoperative dialysis phase and after transplantation, which may result in decreased QoL and have huge burdens on patients. Promoting ideal PA levels among these patients may be a potential approach to improve their physiological function and QoL.

Physical activity interventions pre- or post- surgery have been suggested to optimize the outcomes following abdominal operations (9, 10). Continuous exercise interventions have also been shown to improve physical function and health-related QoL in dialysis patients with an increased PA level (11). In the early postoperative period, physiotherapy protocols also contribute to better muscle strength, aerobic capacity, and QoL in KTRs (12). As an important part of multimodal surgical rehabilitation management, matching the concept of prerehabilitation and enhancing physical activity and function before the operation, has been recognized worldwide (13). Thus, we assume that the PA level of KTRs during pretransplant phase may be inadequate, and KTRs with a higher PA level may perform a better postoperative mobilization and therapeutic efficacy. This study aims to investigate the preoperative PA level of KTRs and analyze the effect of different preoperative PA levels on early postoperative recovery to provide guidance for prehabilitation expansion during KTRs.

## Materials and methods

### Setting and participants

The retrospective study was performed in West China Hospital, Sichuan University. Ethics approval for the study was obtained from the West China Hospital Clinical Trials and Biomedical Ethics Committee of Sichuan University [approval number: 2020 (771)]. Our study was conducted in conformity with the Declaration of Helsinki, and each patient provided written consent before entry.

The inclusion criteria included age between 18 and 65 years; receiving dialysis for more than three months, conforming to the criteria of kidney transplantation; and hospitalized and received kidney transplantation. The exclusion criteria included receiving kidney retransplantation or combined multiple organ transplantation; having limited motor function caused by neuromuscular disease or trauma before the operation; and being unable to complete the assessment or the early rehabilitation due to various reasons, such as cognition, language, etc. Regarding the sample size, we have referred to other relevant studies that investigated PA levels in patients with diseases such as CKD (14) and hemodialysis (15) owing to the lack of similar studies in this research field, and we finally collected 113 patients' records from August to November 2021.

### Allocation criterion

All patients were assessed by the Chinese version of the International Physical Activity Questionnaire-Long Version (IPAQ-LV) (16). PA levels related to work, transportation, household duties, and leisure time in the week before admission were investigated independently by a professional physical therapists(with more than 5 years experience of early rehabilitation post surgery) after the patients completed the admission procedure and before operation. In addition, the duration time of each intensity in the four types of PA was recorded, and the PA level was converted to the MET value based on the calculation formula specified in the previous criterion.

Based on the preoperative PA level, the patients were allocated into two groups:

– Patients with moderate or high PA levels (Group MH), patients who reported vigorous PA levels (e.g., running for more than 30 min) in the past three days or more, or moderate PA levels (e.g., walking for at least 30 min) for more than five days during the last week before admission. In addition, patients who reported walking in moderation and who had vigorous PA levels for five days or more and PA ≥ 600 METs * min/week were also included in Group MH.
– Patients with low PA levels (Group L), who reported no PA, or whose PA level during the last week before admission did not reach the criterion of Group MH.

### Outcome measures

#### Early mobilization after the operation

As an essential aspect of enhanced recovery after surgery (ERAS) pathways (17), early mobilization, especially postoperative ambulation activity, has been proven to be a practical approach for reducing respiratory, thromboembolic, and other postoperative complications associated with bed rest (18, 19).

In this study, all patients received a standard postoperative rehabilitation session based on the 2018 Chinese guideline of enhanced recovery management in the perioperative period of kidney transplantation (20) and consists of position shifts, exercise in bed, transfer, and ambulatory training. All rehabilitation sessions were performed in the ward with the possible assistance of professional physical therapists. The results of early postoperative mobilization were recorded as the time patients spent transferring out of bed and the distance patients could reach during ambulatory training on the second postoperative day. These two early mobilization indicators from the patients' data were used as the primary outcomes.

#### Estimated glomerular filtration rate (eGFR) and delayed graft function (DGF)

The eGFR is a widely accepted symbolic indicator for assessing postoperative kidney function in clinical practice and research. Considering its clinical significance, the eGFR of patients from laboratory examination results on postoperative days 1, 3, and 5 was recorded as a secondary outcome. DGF, another secondary outcome and one of the most common early postoperative complications, is associated with poor graft outcomes and high rejection rates (21). This study defines DGF as the need for at least one dialysis treatment or a serum creatinine level that is sustained ≥400 µmol/L within the first postoperative week.

#### Infection and thrombosis

Both respiratory and urinary tract infections are common postoperative complications among KTRs and are related to poor clinical prognosis. Modified perioperative management, such as breathing exercises, early mobilization, and early extubation, has been proven to reduce infections in patients after surgery. However, KTRs still face a high risk of infection, especially in the early postoperative period (22, 23). In addition, thrombosis, especially deep vein thrombosis (DVT), is a severe postoperative complication associated with immobility and may lead to fatal adverse events. The incidence of thrombosis after surgery is also considered an important indicator of patient management in the hospital. These meaningful data of patients can be obtained from the hospital medical records database.

#### Postoperative length of stay (LOS) and unscheduled readmission

In this study, the LOS from operation to hospital discharge or referral to the medical ward was recorded as the postoperative LOS. The unscheduled readmission of patients within three months after discharge was also recorded as a secondary outcome.

### Statistical analysis

Statistical analysis was performed by using SPSS 25.0 statistical software by a blinded researcher. Quantitative variables are presented as the mean ± standard deviation (M ± SD) or the median and interquartile range (M ± IQR) depending on the normal distribution results measured by the Kolmogorov–Smirnov test. Categorical variables are presented as the number (n) or percentage (%). Spearman association analysis was applied to analyze the association among preoperative PA level, early mobilization, and other postoperative recovery indicators. To compare quantitative variables between Group MH and Group L, Student's t test was performed for the data with a normal distribution, and a Mann–Whitney U test was conducted for the variables with a nonnormal distribution. To compare the difference in categorical variables between the two groups, a chi-square test was used. Statistically significant differences were considered if the error probability was less than 5% (*P* < 0.05).

## Results

### Baseline characteristics and the PA level

**Figure 1:**
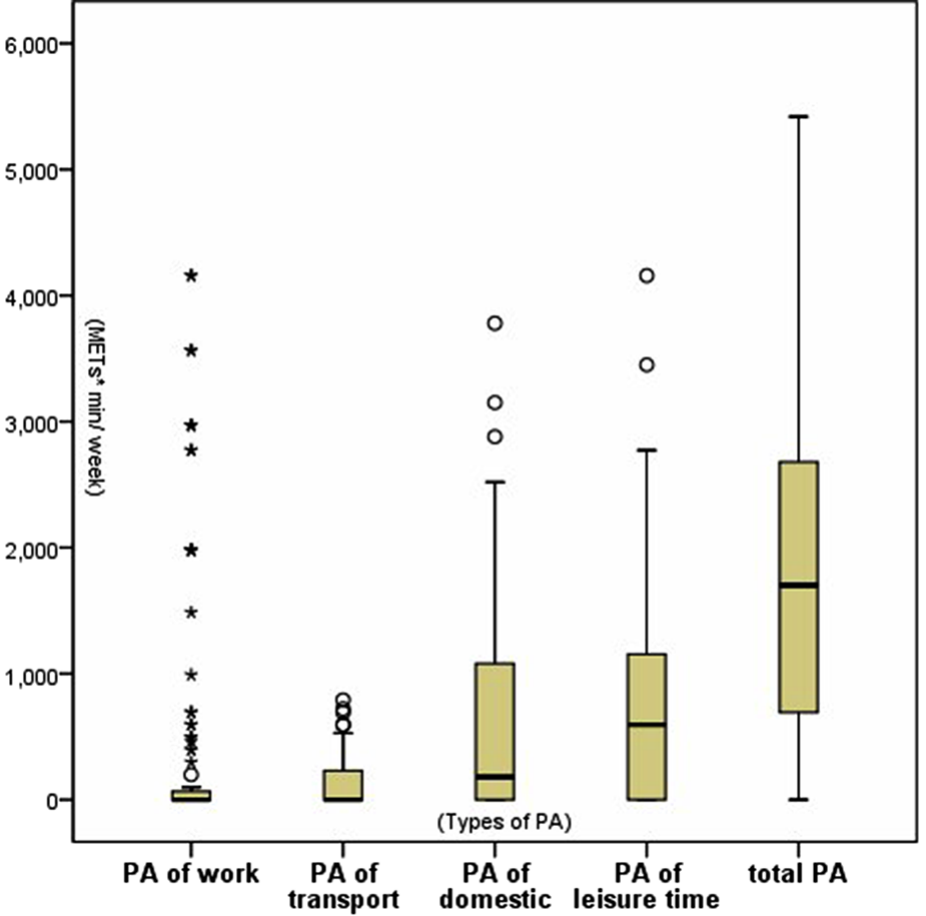
Preoperative PA level of 113 KTRs.

**Figure 2:**
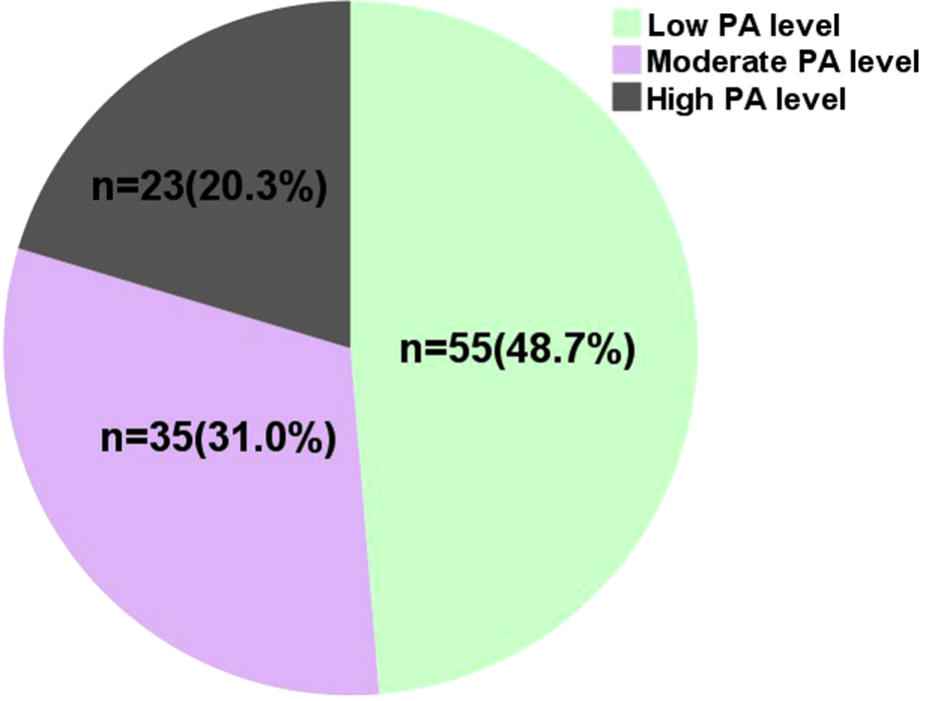
Distribution of PA levels among 113 patients.

As a result of IPAQ-LV, the preoperative PA levels of the 113 KTRs are shown in Figure 1. The median PA level was 1701.0 MTEs * min/week (0 MTEs * min/week for work and transportation, 180.0 MTEs * min/week for household duties, and 594.0 MTEs * min/week for leisure time). As shown in Figure 2, 55 KTRs were categorized into Group L, while 58 KTRs were allocated into Group MH (35 moderate PA level and 23 high) according to their preoperative PA level. There was no significant between-group difference in the baseline characteristics, including age, sex, dialysis age, and types of donors, as shown in Table 1.

**Table 1:** Descriptive characteristics of KTRs (*n* = 113).

	Group L (*n* = 55)	Group MH (*n* = 58)	*χ*^2^/*t*	*P* value
Age, years (mean ± SD)	34.44 ± 11.38	34.85 ± 9.84	0.204	0.838
Gender, *n* (%)			0.030	0.863
Male	35 (63.6)	36 (62.1)		
Female	20 (36.4)	22 (37.9)		
BMI (mean ± SD)	20.62 ± 2.84	20.92 ± 2.89	0.551	0.583
Stature, cm (mean ± SD)	166.22 ± 6.79	165.28 ± 7.24	0.713	0.477
Body mass, kg (mean ± SD)	57.21 ± 10.03	57.25 ± 9.22	0.023	0.982
Dialysis age, months [median (IQR)]	24 (11,40)	19 (12,39)	0.095	0.924
Donor type, *n* (%)			0.178	0.673
Living donor	39 (70.1)	39 (67.2)		
deceased kidney donor	16 (29.1)	19 (32.8)		

KTRs, kidney transplant recipients; Group L, low PA level group; Group MH, medium to high PA level group; SD, standard deviation; BMI, body mass index; IQR, interquartile range.

### Early postoperative mobilization

As shown in Table 2, 113 KTRs spent a mean time of 19.63 h transferring out of bed after surgery, while the KTRs in Group L and Group MH spent a mean time of 19.63 h and 19.53 h, respectively. The difference between the two groups was not significant (*P* = 0.952). Regarding the distance the patients could reach during ambulatory training on the second postoperative day, all 113 patients reached a mean distance of 183.1 m, while the KTRs in Group L and Group MH reached a mean distance of 228.79 m and 134.91 m, respectively. The between-group analysis showed a significant difference between the two groups (*P* < 0.001).

**Table 2:** Postoperative mobilization of KTRs (*n* = 113).

	All KTRs (*n* = 113)	Group L (*n* = 55)	Group MH (*n* = 58)	*t*	d	*P* value
Time spent to achieve transferring out of bed post operation, h (mean ± SD)	19.63 ± 7.54	19.67 ± 7.03	19.59 ± 8.05	0.061	0.011	0.952
Best distance during ambulatory training in the first two days post operation, m (mean ± SD)	183.10 ± 102.55	134.91 ± 92.57	228.79 ± 90.44	5.453	1.026	<0.001

KTRs, kidney transplant recipients; Group L, low PA level group; Group MH, medium to high PA level group; SD, standard deviation.

### Postoperative complications, eGFR, postoperative LOS, and unscheduled readmission

As shown in Table 3, the results revealed a significant between-group difference in postoperative complications. Nine KTR patients were diagnosed with DGF: six were in Group L, and three were in Group MH (*P* = 0.436). Twelve KTR patients who were included were found to have postoperative infections: seven were in Group L, and five were in Group MH (*P* = 0.479). Only one patient in Group L experienced thrombosis of the right femoral vein.

**Table 3:** Postoperative recovery of KTRs (*n* = 113).

	All KTRs (*n* = 113)	Group L (*n* = 55)	Group MH (*n* = 58)	χ^2^/*t*	*d*	*P* value
**Postoperative complications**						
DGF, *n* (%)	9 (8.0)	6 (10.9)	3 (5.2)	0.606	0.147	0.436
Infections, *n* (%)	12 (10.6)	7 (12.7)	5 (8.6)	0.502	0.134	0.479
Thrombosis, *n* (%)	1 (0.9)	1 (1.8)	0	–	–	–
**Postoperative eGFR (ml/min/1.732 m^2^) (mean ± SD)**						
POD1	14.11 ± 9.07	14.02 ± 8.10	14.20 ± 9.99	0.110	0.021	0.913
POD3	46.69 ± 26.42	44.22 ± 24.68	49.04 ± 27.98	0.968	0.182	0.335
POD5	62.00 ± 29.01	60.20 ± 28.19	63.70 ± 29.90	0.640	0.120	0.524
Postoperative LOS, days (mean ± SD)	11.38 ± 4.62	12.11 ± 5.71	10.69 ± 3.19	1.643	0.309	0.103
Unscheduled readmission in three months after discharge, *n* (%)	15 (13.3)	8 (14.5)	7 (12.1)	0.150	0.073	0.698

KTRs, kidney transplant recipients; Group L, low PA level group; Group MH, medium to high PA level group; DGF, delayed graft function; eGFR, estimated glomerular filtration rate; SD, standard deviation; LOS, length of stay.

The eGFR levels on postoperative days 1, 3, and 5 in both groups were comparable with no significant difference (*P* = 0.913, 0.335, and 0.524). The mean postoperative LOS for all KTRs included was 11.38 d, it was 12.11 d for Group L and it was 10.69 d for Group MH. However, no obvious between-group differences were found in the above indicator (*P* = 0.103). A total of 15 unscheduled readmissions in the three months after discharge were recorded: eight for Group L and seven for Group MH, with no significant difference between the two groups (*P* = 0.698).

### Associations between the preoperative PA level and early mobilization and other postoperative recovery indicators

According to the Spearman association analysis, the preoperative PA level showed a moderate positive association with early postoperative ambulation distance (*ρ* = 0.497, *P* < 0.001). Moreover, no significant association was found between the preoperative PA level and other postoperative recovery indicators(e.g., eGFR, infections, DGF, postoperative LOS and unscheduled readmission). The time patients spent transferring out of bed after the operation showed relatively low negative associations with early postoperative ambulation distance (*ρ *= −0.237, *P* = 0.011) and postoperative LOS (*ρ* = −0.298, *P* = 0.001).

## Discussion

In the present study, the median PA level of 113 KTRs before the operation was 1701.0 METs * min/week with the quartile spacing from 693.0 METs * min/week to 2689.5 METs * min/week. We noticed that patients with CKD during the dialysis stage had a lower PA level than the general population, but they still showed a higher PA level than patients undergoing hemodialysis or peritoneal dialysis, as reported by Liu Yanping (24) and Zhang Yue (25). We suppose that KTRs need a relatively higher PA level during the candidate phase may to maintain qualification for transplantation indications because the low PA level is associated with CVD, frailty, and infection. Some KTR candidates might be disqualified due to contraindications associated with low PA levels and were not included in this study, leading to the conclusion that KTRs show a relatively higher PA level than patients undergoing hemodialysis or peritoneal dialysis.

In terms of the composition of preoperative PA levels in the 113 KTRs, only a few patients (*n* = 29, 25.7%) reported PA levels related to work, which corresponded to the low employment rate of dialysis patients. Kirkeskov L (26) reported that the employment rate among dialysis patients is 26.3% for the weighted mean ranging from 10.5% to 59.7%. In the United States, the employment rate among patients on dialysis is 23%–24%, and 38% of all these patients stop working after dialysis initiation (27). In China, the employment rate is 50.65% before dialysis and quickly declines to 22.2% in the first dialysis year with no reemployment. The reasons for patients quitting their jobs during dialysis include physical status, time spent on dialysis, lack of support or acceptance from employers and family, and resistance from family, as reported by Huang B (28). Thus, improving the employment status during the candidate phase before transplantation may be a potential way to improve the socioeconomic status and multiple dimensions of QoL in KTRs before the operation.

Early mobilization, a vital part of ERAS pathways, has been widely applied in postoperative patients. As Zhu Q (29) reported, an early mobilization intervention led to a 1.6-day shorter duration of the indwelling drainage tube and a 3.4-day reduced LOS after kidney transplantation. From a retrospective study of Dias BH (30), an early postoperative mobilization protocol for KTRs is feasible, cost-saving, and two days shorter in LOS. In this study, the time spent by patients to achieve transfer out of bed was associated with a short postoperative LOS. Nevertheless, this value was approximate in both groups with no significant difference. The distance during the postoperative ambulatory training showed a relatively low negative association with time spent transferring out of bed and a positive association with preoperative PA level. The distance the KTRs in Group MH reached in the postoperative ambulatory training was nearly 1.7 times that of the KTRs in Group L, with a significant difference. Transferring may be a relatively lower intensity exercise during the early postoperative rehabilitation session. With available assistance offered by physical therapists, most KTRs can transfer out of bed. However, ambulation may be relatively more challenging than transferring exercise; KTRs may need more physical fitness restoration and more clinical conditions to deal with postoperative ambulation under the acquired assistance. KTRs with higher PA levels before the operation may have more surgical tolerance, physical fitness storage, and a stronger willingness to exercise. Thus, they need to be advised to perform early postoperative mobilization, especially in energy-consuming activities such as ambulatory training.

As a systematic review of Hijazi (31) shows, prehabilitation programs including physical exercise improves patient`s exercise capacity and health-related quality of life but postoperative complications after abdominal cancer surgery. From a *post hoc* analysis of two randomized trials by Rivas E (32), better early mobilization after the abdominal operation was associated with lower pain scores and fewer complications. Meanwhile, in a randomized controlled trial performed by de Almeida EPM (33), patients receiving major abdominal cancer surgery with an early mobilization program showed better functional capacity and QoL, but no between-group difference regarding postoperative complications assessed by the Clavien–Dindo classification was found. In this study, the between-group analysis showed no significant difference in the clinical outcomes, including eGFR, postoperative complications (e.g., DGF, respiratory and urinary tract infections), postoperative LOS, and unscheduled readmission within three months after discharge. Improved postoperative clinical outcomes, such as reduced complications, shortened LOS, and less readmissions, may result from a multidimensional modification, which includes the operation skill, nursing, medicine, and management concept in the perioperative period. With the cooperation of a multidisciplinary medical team, KTRs might achieve better postoperative recovery and overcome some obstacles by themselves. Thus, the PA level before the operation might not be associated with some clinical outcomes to a certain extent.

This study is limited to a single-center retrospective study with a small sample size over a short period. Due to a lack of similar literature, our sample size can only refer to other similar studies, which may also lead to some bias. The majority of the results in this study were collected from the digital electronic medical record system, and some established postoperative outcome measures, e.g., comprehensive complications index for postoperative complications was not applied, and only the occurrence of few most common postoperative complications was observed. Besides, This study may also have been biased due to differences in the pathophysiology of the KTRs during ESRD phase (the autoimmune ones for younger population of ESRD patients, and the more chronic ESRD which arise from poorly controlled hypertension/diabetes mellitus for the elder populations). And the readmission rate might be underestimated due to some of the patients having readmission to another hospital without networking with us. Meanwhile, only a better ambulatory distance during postoperative mobilization was observed in Group MH, and the effect size of most comparison is not satisfied. Further studies need to explore more details about PA levels, including preoperative PA levels and their effect on postoperative recovery, with a large sample sizes and better research designs. And we strongly suggest a prehabilitation programme for patients awaiting Kidney transplantation to help them achieve a higher physical activity level before kidney transplantation.

## Conclusion

The preoperative PA level of KTRs is lower than that of the general population but higher than that of patients on dialysis. KTRs with moderate or high PA levels before the operation showed better early mobilization in the early postoperative days than KTRs with low PA levels, but no significant advantage in other recovery indicators (e.g., eGFR, infections, DGF, postoperative LOS and unscheduled readmission) was found. Moreover, future research should explore the potential mechanism and practical application of different PA levels in the early rehabilitation of KTRs.

## Data Availability

The raw data supporting the conclusions of this article will be made available by the authors, without undue reservation.
