# Supplementary material for: Investigation of preoperative physical activity level in kidney transplant recipients and its impact on early postoperative recovery: A retrospective cohort study
Source: Front Surg. 2023 Jan 6;9:1062652. doi: 10.3389/fsurg.2022.1062652 (PMC9852711; doi:10.3389/fsurg.2022.1062652)
Supplement: Supplementary file 1 [file Table1.docx]

*Table 1. Descriptive characteristics of KTRs（n=113）*

|  | *Group L (n=55)* | *Group MH (n=58)* | *χ2/t* | *P value* |
| --- | --- | --- | --- | --- |
| *Age, years(mean±SD)* | *34.44±11.38* | *34.85±9.84* | *0.204* | *0.838* |
| *Gender, n(%)* |  |  | *0.030* | *0.863* |
| *Male* | *35(63.6)* | *36(62.1)* |  |  |
| *Female* | *20(36.4)* | *22(37.9)* |  |  |
| *BMI(mean±SD)* | *20.62±2.84* | *20.92±2.89* | *0.551* | *0.583* |
| *Stature,cm(mean±SD)* | *166.22±6.79* | *165.28±7.24* | *0.713* | *0.477* |
| *Body mass,kg(mean±SD)* | *57.21±10.03* | *57.25±9.22* | *0.023* | *0.982* |
| *Dialysis age, months[median(IQR)]* | *24(11,40)* | *19(12,39)* | *0.095* | *0.924* |
| *Donor type, n(%)* |  |  | *0.178* | *0.673* |
| *Living donor* | *39(70.1)* | *39 (67.2)* |  |  |
| *deceased kidney donor* | *16(29.1)* | *19(32.8)* |  |  |
| *KTRs= kidney transplant recipients, Group L= low PA level group, Group MH= medium to high PA level group , SD= standard deviation, BMI= body mass index, IQR= interquartile range.* | | | | |

*Table 2. Postoperative mobilization of KTRs（n=113）*

|  | *All KTRs (n=113)* | *Group L (n=55)* | *Group MH (n=58)* | *t* | *d* | *P value* |
| --- | --- | --- | --- | --- | --- | --- |
| *Time spent to achieve transferring out of bed post operation, h(mean±SD)* | *19.63±7.54* | *19.67±7.03* | *19.59±8.05* | *0.061* | *0.011* | *0.952* |
| *Best distance during ambulatory training in the first two days post operation, m(mean±SD)* | *183.10±102.55* | *134.91±92.57* | *228.79±90.44* | *5.453* | *1.026* | *＜0.001* |
| *KTRs= kidney transplant recipients, Group L= low PA level group, Group MH= medium to high PA level group , SD= standard deviation.* | | | | | | |

*Table 3. Postoperative recovery of KTRs（n=113）*

|  | *All KTRs (n=113)* | *Group L (n=55)* | *Group MH (n=58)* | *χ2/t* | *d* | *P value* |
| --- | --- | --- | --- | --- | --- | --- |
| *Postoperative complications* |  |  |  |  |  |  |
| *DGF, n(%)* | *9(8.0)* | *6(10.9)* | *3(5.2)* | *0.606* | *0.147* | *0.436* |
| *Infections, n(%)* | *12(10.6)* | *7(12.7)* | *5(8.6)* | *0.502* | *0.134* | *0.479* |
| *Thrombosis, n(%)* | *1 (0.9)* | *1(1.8)* | *0* | *-* | *-* | *-* |
| *Postoperative eGFR,*  *(ml/min/1.732 m^2)(mean±SD)* |  |  |  |  |  |  |
| *POD1* | *14.11±9.07* | *14.02±8.10* | *14.20±9.99* | *0.110* | *0.021* | *0.913* |
| *POD3* | *46.69±26.42* | *44.22±24.68* | *49.04±27.98* | *0.968* | *0.182* | *0.335* |
| *POD5* | *62.00±29.01* | *60.20±28.19* | *63.70±29.90* | *0.640* | *0.120* | *0.524* |
| *Postoperative LOS,*  *days(mean±SD)* | *11.38±4.62* | *12.11±5.71* | *10.69±3.19* | *1.643* | *0.309* | *0.103* |
| *Unscheduled readmission in three months after discharge, n(%)* | *15(13.3)* | *8 (14.5)* | *7 (12.1)* | *0.150* | *0.073* | *0.698* |
| *KTRs= kidney transplant recipients, Group L= low PA level group, Group MH= medium to high PA level group , DGF= delayed graft function, eGFR= estimated glomerular filtration rate, SD= standard deviation, LOS= length of stay.* | | | | | | |
